# Acute Hemodynamic Effect of Acetazolamide in Patients With Pulmonary Hypertension Whilst Breathing Normoxic and Hypoxic Gas: A Randomized Cross-Over Trial

**DOI:** 10.3389/fmed.2021.681473

**Published:** 2021-07-22

**Authors:** Mona Lichtblau, Charlotte Berlier, Stéphanie Saxer, Arcangelo F. Carta, Laura Mayer, Alexandra Groth, Patrick R. Bader, Simon R. Schneider, Michael Furian, Esther I. Schwarz, Erik R. Swenson, Konrad E. Bloch, Silvia Ulrich

**Affiliations:** ^1^Clinic of Pulmonology, University Hospital Zurich, Zurich, Switzerland; ^2^Division of Pulmonary, Critical Care and Sleep Medicine, VA Puget Sound Health Care System, University of Washington, Seattle, WA, United States

**Keywords:** acetazolamide, pulmonary arterial hypertension, hemodynamics, hypoxia, normoxia, right heart catheterization, pulmonary vascular disease

## Abstract

**Aims:** To test the acute hemodynamic effect of acetazolamide in patients with pulmonary hypertension (PH) under ambient air and hypoxia.

**Methods:** Patients with pulmonary arterial or chronic thromboembolic PH (PAH/CTEPH) undergoing right heart catheterization were included in this randomized, placebo-controlled, double-blinded, crossover trial. The main outcome, pulmonary vascular resistance (PVR), further hemodynamics, blood- and cerebral oxygenation were measured 1 h after intravenous administration of 500 mg acetazolamide or placebo-saline on ambient air (normoxia) and at the end of breathing hypoxic gas (F_I_O_2_ 0.15, hypoxia) for 15 min.

**Results:** 24 PH-patients, 71% men, mean ± SD age 59 ± 14 years, BMI 28 ± 5 kg/m^2^, PVR 4.7 ± 2.1 WU participated. Mean PVR after acetazolamide vs. placebo was 5.5 ± 3.0 vs. 5.3 ± 3.0 WU; mean difference (95% CI) 0.2 (−0.2–0.6, *p* = 0.341). Heart rate was higher after acetazolamide (79 ± 12 vs. 77 ± 11 bpm, *p* = 0.026), pH was lower (7.40 ± 0.02 vs. 7.42 ± 0.03, *p* = 0.002) but PaCO_2_ and PaO_2_ remained unchanged while cerebral tissue oxygenation increased (71 ± 6 vs. 69 ± 6%, *p* = 0.017). In acute hypoxia, acetazolamide decreased PVR by 0.4 WU (0.0–0.9, *p* = 0.046) while PaO_2_ and PaCO_2_ were not changed. No adverse effects occurred.

**Conclusions:** In patients with PAH/CTEPH, i.v. acetazolamide did not change pulmonary hemodynamics compared to placebo after 1 hour in normoxia but it reduced PVR after subsequent acute exposure to hypoxia. Our findings in normoxia do not suggest a direct acute pulmonary vasodilator effect of acetazolamide. The reduction of PVR during hypoxia requires further corroboration. Whether acetazolamide improves PH when given over a prolonged period by stimulating ventilation, increasing oxygenation, and/or altering vascular inflammation and remodeling remains to be investigated.

## Introduction

Precapillary pulmonary hypertension (PH) is characterized by chronic elevation of pulmonary artery pressure (PAP) and pulmonary vascular resistance (PVR) due to reduction and/or remodeling of the pulmonary vascular bed in the absence of elevated left atrial pressure and hypoxaemic lung disease. Major forms are pulmonary arterial and chronic thromboembolic PH, hereafter summarized as PH (1). The leading symptom in PH is dyspnoea on exertion leading to exercise limitation, reduced daily activity and impaired quality of life (2–4). Exertional and sleep-related hypoxaemia (5) are further consequences of PH that may impair daytime performance (5–7). The prognosis of untreated PH is poor as it progressively leads to right heart failure and death within months to a few years (8). Recent therapeutic advances including medical and interventional treatments have improved life expectancy, physical performance and quality of life in PH-patients (9–12). However, PH is still incurable and many patients have persistent impairments despite optimized treatment. Therefore, new therapeutics that act on different pathways are needed.

One of the potential candidate drugs that have not been conclusively evaluated for clinical use in patients with PH is acetazolamide, a carbonic anhydrase inhibitor, that stimulates ventilation by inducing a metabolic acidosis via renal bicarbonate excretion and this effect is especially pronounced in a hypoxic environment (13). In healthy mountaineers ascending to high altitude, acetazolamide improves oxygenation and is effective in prevention of acute mountain sickness. (14) It has also been suggested that acetazolamide may mitigate the excessive rise in PAP observed in individuals susceptible to high altitude pulmonary edema when exposed to hypoxia. (15, 16) However, the time-lag from oral acetazolamide administration to the increased ventilation induced by metabolic acidosis from sufficiently increased bicarbonate excretion requires several hours. (13) Of interest for patients with PH and mountaineers at risk of PH in a hypoxic environment, recent studies have revealed that acetazolamide may cause pulmonary vasodilatation independent of CA inhibition (17), which opens up the possibility that a drug targeting this mechanism might be useful without causing the known side effects of carbonic anhydrase inhibition. Moreover, data from physiologic studies in isolated pulmonary smooth muscle cells of rats and in awake dogs as well as preliminary observations in humans have suggested that acetazolamide may cause pulmonary vasodilatation independent of renal carbonic anhydrase inhibition, in particular, in the presence of hypoxia (17–22). In a randomized, placebo-controlled clinical trial in patients with PH we have previously shown that acetazolamide administered orally over the course of 1 week improved nocturnal arterial oxygen saturation and sleep disordered breathing but the study was not designed to evaluate effects on pulmonary hemodynamics (23).

To further investigate the potential actions of acetazolamide on the pulmonary circulation, the objective of the current study was to test the hypothesis that acetazolamide reduces pulmonary vascular resistance (PVR) and that this effect would be especially pronounced whilst breathing hypoxic gas mixtures (normobaric hypoxia). By administering acetazolamide intravenously and focusing on the acute (within 1 h) hemodynamic effects assessed during right heart catheterisation (RHC) we sought to determine whether acetazolamide would exert a direct pulmonary vasodilatory effect independent of renal carbonic anhydrase inhibition and development of the typical drug-induced mild metabolic acidosis and that this effect would be especially pronounced whilst breathing normobaric hypoxia.

## Methods

### Study Design and Subjects

This was a randomized, placebo-controlled, double-blind, cross-over trial assessing the acute effects of acetazolamide in patients with precapillary PH undergoing RHC for clinical reasons. The protocol comprised of supine resting hemodynamic measurements by RHC at 3 consecutive time points (a) baseline measurements (b) 60 min after 500 mg acetazolamide i.v. and (c) 60 min after placebo-saline i.v. injections, b and c in a double-blinded, randomized order. After resting measurements in periods b and c patients breathed normobaric hypoxic gas (FiO_2_ 0.15) through an airtight mouthpiece (AltiTrainer, SMTEC, Nyon, Switzerland) for a duration of 10–15 min, at the end of which measurements were repeated whilst breathing normobaric hypoxic gas 80–90 min. after acetazolamide vs. placebo injection. All patients gave written informed consent. The study complies with the declaration of Helsinki and was approved by the local ethical authorities (BASEC 2016-00089) and registered at clinicaltrial.gov (NCT02755259).

Individuals referred for clinically indicated RHC fulfilling the following inclusion criteria were eligible: signed informed consent, NYHA functional class I-IV, age 20–80 years, both sexes, precapillary PH diagnosed according to guidelines (mPAP ≥ 25 mmHg along with a PAWP ≤ 15 mmHg) 0.2 (24). Patients were excluded if one of the following was present: PH due to left heart disease, at least moderate chronic obstructive pulmonary disease (FEV_1_ ≤ 60% pred.), or restrictive lung disease (FVC ≤ 60% pred.), severe daytime hypercapnia (pCO_2_ > 6.5 kPa), liver disease, non-correctable electrolyte disturbances or severe chronic kidney and liver disease, pregnancy or breast feeding and known allergic response to acetazolamide and other known carbonic anhydrase inhibitors; methazolamide, dichlorphenamide, thiazide diuretics, and sulfonamide loop diuretics.

### Randomization, Intervention and Blinding

Patients were randomized in balanced blocks of four to one of the two sequences (placebo/acetazolamide or acetazolamide/placebo) by a computer program. Participants and investigators were blinded to the administered drug until the conclusion of data analysis.

### Assessments

#### Right Heart Catheterization

A Swan-Ganz catheter (Swan Ganz CCOmbo V, Edwards Lifesciences, Irvine, CA 92614, USA) was placed in the pulmonary artery via the right jugular vein under sonographic control. Transducers were set at the midthoracic level and zeroed to atmospheric pressure (25). The following parameters were measured and averaged over several respiratory cycles: heart rate (HR), pulmonary arterial pressure (PAP systolic, diastolic, mean), pulmonary arterial wedge pressure (PAWP), and right atrial pressure (RAP) 0.2 (26). Cardiac output (CO) was measured by thermodilution in triplicate by cold saline injection (Vigilance II, Edwards Lifesciences, Irvine CA 92614, USA). Cardiac index (CI) was calculated as CO/body surface area. PVR was calculated as PVR = (mPAP–PAWP)/CO.

#### Blood Gas Analysis and Oximetry

A radial artery catheter was placed for continuous blood pressure monitoring and arterial blood gas sampling. Arterial and mixed venous blood gases were drawn from the arterial line and the distal port of the Swan Ganz catheter, respectively, and immediately analyzed (ABL 90 Flex-blood analyser, Radiometer GmbH). Pulse oximetric oxygen saturation was continuously monitored.

#### Additional Continuous Non-invasive Assessments

Frontal cerebral and quadriceps muscle tissue oxygenation were measured by near infrared spectroscopy (NIRS, NIRO 200NX, Hamamatsu Photonics SA, Hamamatsu, Japan). NIRS-optodes were fixed to the forehead and quadriceps muscle by adhesive tape and secured by elastic banding as described (27, 28).

## Outcomes

The main outcome of this study was the difference in resting PVR measured 60 min. after acetazolamide vs. placebo administration at ambient air. An additional main outcome was the change in PVR induced by hypoxia after acetazolamide vs. placebo. Secondary outcomes were other hemodynamic and oxygenation measures including the mPAP, CO, CI, RAP, PAWP, systemic blood pressure, arterial and mixed venous blood gases analysis including pH and bicarbonate, and tissue oxygenation, and the changes of these measures during breathing hypoxia after acetazolamide vs. placebo.

### Sample Size

It was estimated that 18 participants would be needed to detect an assumed clinically important difference in PVR of 1.5 WU (SD 1.5 WU) with a two-sided significance level of 0.05 and a power of 80 %.

### Statistical Analysis

The data are summarized as means and standard deviation. The outcomes were analyzed in the intention-to-treat population using a mixed multivariable regression modeling including interventions (placebo or acetazolamide and normoxia or hypoxia) and order of administration as independent variables and are given as mean differences and 95% confidence interval (CI) as computed by the regression analysis. Since there were no missing values in hemodynamic measurements, no imputation was necessary. The treatment effects were also calculated by additionally adjusting for respective baseline values, age, sex, and PH-group (PAH/CTEPH). All statistical analyses were performed using R Studio (version 1.2.1578, R Studio Inc., San Francisco, USA). Statistical significance was assumed when 95% confidence intervals of mean differences did not overlap zero and *p* < 0.05.

## Results

**Figure 1:**
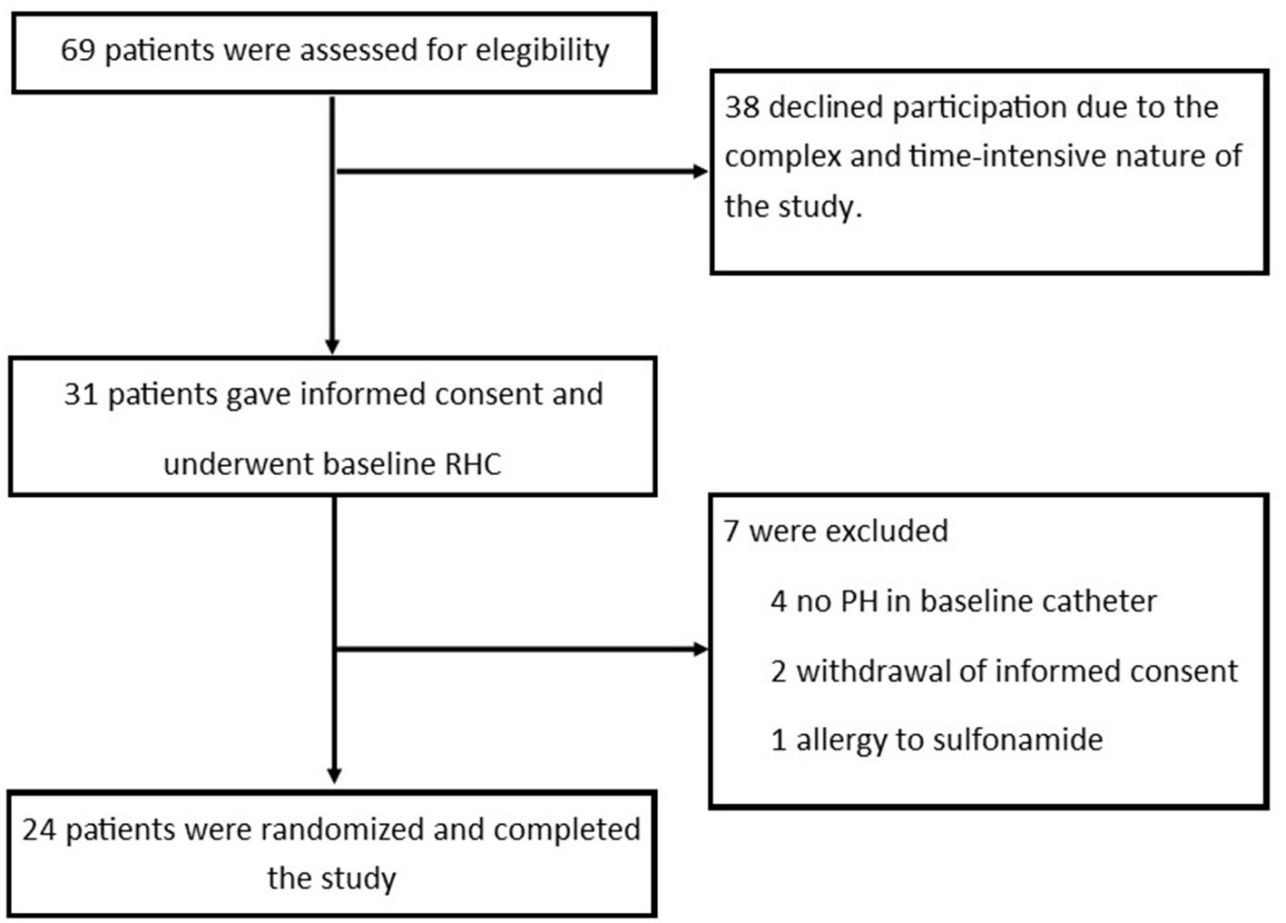
Study flow.

Of 69 patients screened for eligibility, 31 patients gave written informed consent to participate. Seven patients had to be excluded after baseline hemodynamic assessment (four no precapillary PH, two withdrawn consent, one sulfonamide allergy) resulting in 24 PH-patients that were available for randomization (Figure 1). Their characteristics and hemodynamics are shown in Table 1. In brief, they were mostly male (71%), 29% had PAH and 71% CTEPH, most of them were in functional class II (63%) and the mean ± SD age was 59 ± 14 years, mPAP 35 ± 9 mmHg, CI 2.6 ± 0.5 l/min, PVR 4.7 ± 2.1 WU.

**Table 1:** Baseline characteristics.

**Baseline characteristics**	**Number (%) or mean ± SD**
*N* = 24	
Sex (f/m), *n* (%)	7/17 (29 % /71 %)
Age, y	59 ± 14
Body mass index, kg/m^2^	28 ± 5
WHO-functional class, *n* (%)	
I	2 (8%)
II	15 (63%)
III	7 (29%)
Pulmonary hypertension classification	
Idiopathic pulmonary arterial hypertension, *n* (%)	7 (29%)
Chronic thromboembolic pulmonary hypertension, n (%)	17 (71%)
Baseline hemodynamics	
Heart rate, bpm	67 ± 12
Systolic blood pressure, mmHg	133 ± 14
Mean blood pressure, mmHg	93 ± 9
Diastolic blood pressure, mmHg	71 ± 7
Systolic pulmonary artery pressure, mmHg	55 ± 18
Mean pulmonary artery pressure, mmHg	35 ± 9
Diastolic pulmonary artery pressure, mmHg	23 ± 7
Right atrial pressure, mmHg	7 ± 3
Cardiac index, l/min/m^2^	2.6 ± 0.5
Pulmonary artery wedge pressure, mmHg	11 ± 2
Pulmonary vascular resistance, WU	4.7 ± 2.1
SmvO_2_, %	67 ± 8
SpO_2_, %	95 ± 2
6-min walking distance, m	531 ± 111
SpO_2_ end 6 MWD, %	90 ± 7
NT-proBNP, ng/l	485 ± 812
FEV_1_, % predicted	94 ± 17
FVC, % predicted	95 ± 17
TLC, % predicted	96 ± 17
D_L_CO, % predicted	83 ± 28

*PH, pulmonary hypertension, SmVO_2_, mixed venous oxygen saturation, SpO2, oxygen saturation by pulseoximetry, FEV_1_, forced expiratory volume in 1 s, FVC, forced vital capacity, TLC, total lung capacity, DLCO, diffusion lung capacity for carbon monoxide*.

### Measurements 60 Min After Acetazolamide vs. Placebo Administration

**Figure 2:**
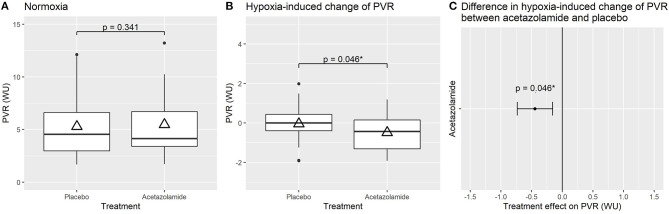
Effect of treatment with acetazolamide vs. placebo on pulmonary vascular resistance (PVR) under ambient air **(A)**, on the hypoxia-induced change in PVR **(B)** and mean difference (95% confidence interval) in hypoxia-induced change between acetazolamide and placebo treatment **(C)**. Acetazolamide did not have a significant effect on PVR under ambient air **(A)**, under hypoxia, PVR remained unchanged with placebo but was significantly reduced by acetazolamide **(B,C)**. Boxplots **(A,B)** with horizontal line = median; upper and lower box edge = interquartile ranges [IQR]; length of whiskers = 1.5 × IQR; dots = outliers; and triangles = means. Statistically significant values (*p* < 0.05 or 95% CI not including 0) are denoted with *.

The primary outcome PVR, was similar after acetazolamide vs. placebo [5.5 ± 3.0 vs. 5.3 ± 3.0, mean difference (95% confidence) 0.2 (−0.2–0.6) WU, *p* = 0.341], (Figure 2, Table 2). The HR was slightly higher after acetazolamide compared to placebo [79 ± 12 vs. 77 ± 11 bpm; 2 (0–4), *p* = 0.026]. There were no significant differences in other hemodynamic parameters between acetazolamide and placebo (Table 2). We also did not find a treatment effect of acetazolamide vs. placebo when the main hemodynamic parameters were corrected for age, sex, respective baseline values, or treatment order (Supplementary Table 1).

**Table 2:** The effect of acetazolamide vs. placebo on invasive pulmonary hemodynamics, blood gases and tissue oxygenation after 60 min under ambient gas.

	**Placebo**	**Acetazolamide**	**Treatment effect**	
**Variable**	**Mean ± SD**	**Mean ± SD**	**Mean difference (95% CI)**	***P* value**
**Main Outcome**				
Pulmonary vascular resistance, WU	5.3 ± 3.0	5.5 ± 3.0	0.2 (−0.2 to 0.6)	0.341
**Resting hemodynamics**				
Heart rate, bmp	77 ± 11	79 ± 12	2 (0 to 4)	0.026^*^
Systolic blood pressure, mmHg	125 ± 13	123 ± 15	−2 (−6 to 3)	0.531
Mean blood pressure, mmHg	87 ± 10	84 ± 10	2 (−5 to 1)	0.115
Diastolic blood pressure, mmHg	67 ± 9	65 ± 8	−2 (−4 to 0)	0.097
Systolic pulmonary artery pressure, mmHg	63 ± 21	62 ± 19	−1 (−5 to 4)	0.780
Mean pulmonary artery pressure, mmHg	39 ± 11	40 ± 11	1 (−1 to 2)	0.517
Diastolic pulmonary artery pressure, mmHg	25 ± 8	25 ± 7	0 (−1 to 2)	0.602
Right atrial pressure, mmHg	8 ± 4	7 ± 3	−1 (−2 to 0)	0.065
Cardiac index, l/(min^*^m^2^)	3.0 ± 0.7	2.9 ± 0.6	−0.1 (−0.3 to 0.1)	0.342
Pulmonary artery wedge pressure, mmHg	11 ± 3	11 ± 3	0 (−1 to 1)	0.851
**Arterial blood gas analysis**				
Arterial pH	7.42 ± 0.03	7.40 ± 0.02	−0.01 (−0.02 to −0.01)	<0.001^*^
Hemoglobin, g/dl	14.4 ± 1.2	14.4 ± 1.4	0.2 (0.0 to 0.3)	0.094
Lactate, mmol/l	1.1 ± 0.5	1.1 ± 0.4	0.0 (−0.2 to 0.1)	0.622
HbO_2_, %	91 ± 3	91 ± 4	0 (−1 to 1)	0.757
PaO_2_, kPa	8.6 ± 1.6	9.0 ± 1.8	0.2 (−0.1 to 0.6)	0.208
PaCO_2_, kPa	4.4 ± 0.5	4.4 ± 0.5	0.0 (−0.1 to 0.1)	0.756
Bicarbonate, mmol/l	22.5 ± 1.5	21.8 ± 1.4	−0.5 (−0.9 to −0.2)	0.004^*^
**Mixed venous blood gas analysis**				
HbmvO_2_, %	63 ± 7	64 ± 6	1 (−1 to 3)	0.277
PmvO_2_, kPa	4.6 ± 0.5	4.7 ± 0.4	0 (0 to 0)	0.200
PmvCO_2_, kPa	5.0 ± 0.5	5.1 ± 0.4	0 (0 to 0)	0.042^*^
**Non-invasive assessments**				
SpO_2_ by pulseoximetry, %	92 ± 4	92 ± 3	0 (−1 to 1)	0.911
SmvO_2_ by catheter tip-oximetry, %	66 ± 8	65 ± 7	−1 (−4 to 2)	0.595
Cerebral tissue oxygenation, %	69 ± 6	71 ± 6	2 (0 to 4)	0.017^*^
Cerebral total hemoglobin concentration, unit	1.0 ± 0.2	1.0 ± 0.2	0.1 (0.0 to 0.2)	0.087
Quadriceps tissue oxygenation, %	74 ± 9	75 ± 9	1 (0 to 2)	0.192
Quadriceps total hemoglobin concentration, unit	1.0 ± 0.1	1.0 ± 0.1	0.0 (0.0 to 0.1)	0.071

*Data are displayed as mean ± SD. Treatment effect is adjusted for order of treatment and displayed as mean difference and 95% confidence interval (95% CI)*.

* *indicates statistical significance between placebo-saline and acetazolamide in the randomized phase*.

*HbO_2_, oxyhaemoglobin, PaO_2_, arterial partial pressure of oxygen, PaCO_2_, arterial partial pressure of carbon dioxide, HbmvO_2_, mixed venous oxyhaemoglobin, PmvO_2_, mixed-venous partial pressure of oxygen, PmvCO_2_, mixed-venous partial pressure of carbon dioxide, SmvO_2_, mixed venous oxygen saturation*.

A significant but small reduction in arterial pH was found after acetazolamide vs. placebo [7.40 ± 0.02 vs. 7.42 ± 0.03; −0.01 (−0.02 to −0.01), *p* < 0.001] and bicarbonate concentration was slightly decreased [21.8 ± 1.4 vs. 22.5 ± 1.5 mmol/l; −0.5 (−0.9 to −0.2), *p* = 0.004]. There were no significant differences in arterial pO_2_, pCO_2_, or oxygen saturation, the mixed-venous pCO_2_ was slightly increased. The cerebral tissue oxygenation was increased after acetazolamide [71 ± 6 vs. 69 ± 6 %; 2 (0–4), *p* = 0.017], while quadriceps tissue oxygenation remained unchanged (Table 2).

### Changes Induced by Breathing Normobaric Hypoxic Gas (F_I_O_2_ 0.15)

Whereas, the PVR did not significantly change by breathing normobaric hypoxic gas for 10–15 min with placebo, the PVR decreased with hypoxic gas breathing after acetazolamide resulting in a significant difference [−0.4 WU (−0.9 to −0.0), *p* = 0.046] accompanied by a non-statistically significant increase in cardiac output. There were no significant differences in other hemodynamic or oxygenation parameters induced by acetazolamide vs. placebo during hypoxic gas breathing except for the arterial pCO_2_, which decreased less after acetazolamide (Table 3, Supplementary Table 2).

**Table 3:** The effect of acetazolamide vs. placebo on invasive pulmonary hemodynamics, blood gases and tissue oxygenation after 60 min under hypoxic gas.

	**Placebo**	**Acetazolamide**	**Placebo**	**Acetazolamide**	**Treatment effect**	
**Assessments**			**Change induced by breathing hypoxic gas vs ambient air**	**Change induced by breathing hypoxic gas vs ambient air**	**Mean difference (95% CI) of the changes**	***p* value**
**Main Outcome**						
Pulmonary vascular resistance, WU	5.2 ± 3.0	5.0 ± 2.8	0.0 ± 1.0	−0.5 ± 0.8^†^	−0.4 (−0.9 to −0.0)	0.046^*^
**Additional resting hemodynamics**						
Heart rate, bpm	80 ± 11	81 ± 11	3 ± 5^†^	2 ± 6	−1 (−3 to 1)	0.283
Systolic blood pressure, mmHg	125 ± 15	124 ± 17	0 ± 8	−1 ± 10	1 (−4 to 6)	0.672
Mean blood pressure, mmHg	87 ± 12	84 ± 11	1 ± 5	0 ± 6	−1 (−3 to 2)	0.558
Diastolic blood pressure, mmHg	68 ± 11	64 ± 9	1 ± 4	−1 ± 5	−1 (−3 to 0)	0.056
Systolic pulmonary artery pressure, mmHg	61 ± 22	62 ± 21	−2 ± 13	0 ± 9	2 (−3 to 7)	0.461
Mean pulmonary artery pressure, mmHg	39 ± 13	39 ± 13	0 ± 7	0 ± 5	0 (−3 to 2)	0.769
Diastolic pulmonary artery pressure, mmHg	25 ± 9	24 ± 8	0 ± 7	−1 ± 5	−2 (−5 to 2)	0.367
Right atrial pressure, mmHg	7 ± 4	6 ± 3	−1 ± 2^†^	−1 ± 2^†^	0 (−1 to 1)	0.445
Cardiac index, l/(min ^*^ m^2^)	3.0 ± 0.7	3.0 ± 0.6	0 ± 0.4	0.2 ± 0.3^†^	0.2 (0.0 to 0.4)	0.111
Pulmonary artery wedge pressure, mmHg	11 ± 3	11 ± 3	0 ± 2	0 ± 1	0 (−1 to 1)	0.601
**Arterial blood gas analysis**						
Arterial pH	7.46 ± 0.05	7.44 ± 0.04	0.05 ± 0.04^†^	0.03 ± 0.03^†^	−0.01 (−0.03 to 0.00)	0.061
Hemoglobin, g/dl	14.0 ± 1.3	14.1 ± 1.2	−0.3 ± 0.5^†^	0.4 ± 0.5^†^	−0.1 (−0.3 to 0.2)	0.680
Lactate, mmol/l	1.1 ± 0.5	1.1 ± 0.4	0 ± 0.2	−0.1 ± 0.2^†^	0.0 (−0.1 to 0.1)	0.442
HbO_2_, %	88 ± 5	87 ± 6	−3 ± 5^†^	−5 ± 5^†^	−1 (−4 to 1)	0.205
PaO_2_, kPa	7.5 ± 1.6	7.3 ± 1.4	−1.2 ± 1.6^†^	−1.7 ± 1.5^†^	−0.5 (−1.1 to 0.1)	0.093
PaCO_2_, kPa	3.9 ± 0.6	4.1 ± 0.6	−0.6 ± 0.6^†^	−0.3 ± 0.5^†^	0.2 (0.0 to 0.5)	0.030^*^
Bicarbonate, mmol/l	22.7 ± 1.7	22.2 ± 1.3	0.4 ± 0.6^†^	0.4 ± 0.5^†^	0.0 (−0.3 to 0.2)	0.948
**Mixed venous blood gas analysis**						
HbmvO_2_, %	62 ± 8	61 ± 7	−2 ± 6	−3 ± 4^†^	−2 (−4 to 1)	0.254
PmvO_2_, kPa	4.3 ± 0.5	4.3 ± 0.4	−0.3 ± 0.4^†^	−0.3 ± 0.3^†^	0.0 (−0.2 to 0.1)	0.703
PmvCO_2_, kPa	4.6 ± 0.5	4.7 ± 0.5	−0.3 ± 0.6^†^	−0.8 ± 1.3^†^	−0.4 (−1.0 to 0.1)	0.122
**Non-invasive assessments**						
SpO_2_ by pulse oximetry, %	90 ± 5	88 ± 5	−3 ± 5^†^	−4 ± 4^†^	−2 (−3 to 0)	0.071
SmvO_2_ cathetertip-oximetry, %	63 ± 9	63 ± 7	−4 ± 5^†^	−2 ± 4^†^	2 (0 to 3)	0.102
Cerebral tissue oxygenation, %	67 ± 7	68 ± 6	−2 ± 4^†^	−3 ± 3^†^	−1 (−3 to 1)	0.268
Cerebral total hemoglobin concentration, unit	1.0 ± 0.3	1.0 ± 0.2	0.1 ± 0.3	0 ± 0.1	−0.1 (−0.2 to 0.1)	0.340
Quadriceps tissue oxygenation, %	73 ± 9	74 ± 9	−1 ± 2^†^	−1 ± 1^†^	0 (−1 to 1)	0.994
Quadriceps total hemoglobin concentration, unit	1.0 ± 0.1	1.1 ± 0.1	0 ± 0	0 ± 0	0.0 (0.0 to 0.0)	0.433

*Data are displayed as mean ± SD and treatment effect as mean difference and 95% confidence interval (95% CI) with data adjusted for order of treatment*.

* *indicates statistical significance between placebo-saline and acetazolamide in the randomized phase*.

† *indicates a statistically significant change whilst breathing hypoxic gas compared to normoxic gas in the respective randomized phase*.

*HbO_2_, oxyhaemoglobin, PaO_2_, arterial partial pressure for oxygen, PaCO_2_, arterial partial pressure of carbon dioxide, HbmvO_2_, mixed venous oxyhemoglobin, PmvO_2_, mixed-venous partial pressure for oxygen, PmvCO_2_, mixed-venous partial pressure for carbon dioxide, SmvO_2_, mixed venous oxygen saturation*.

### Additional Analysis

**Figure 3:**
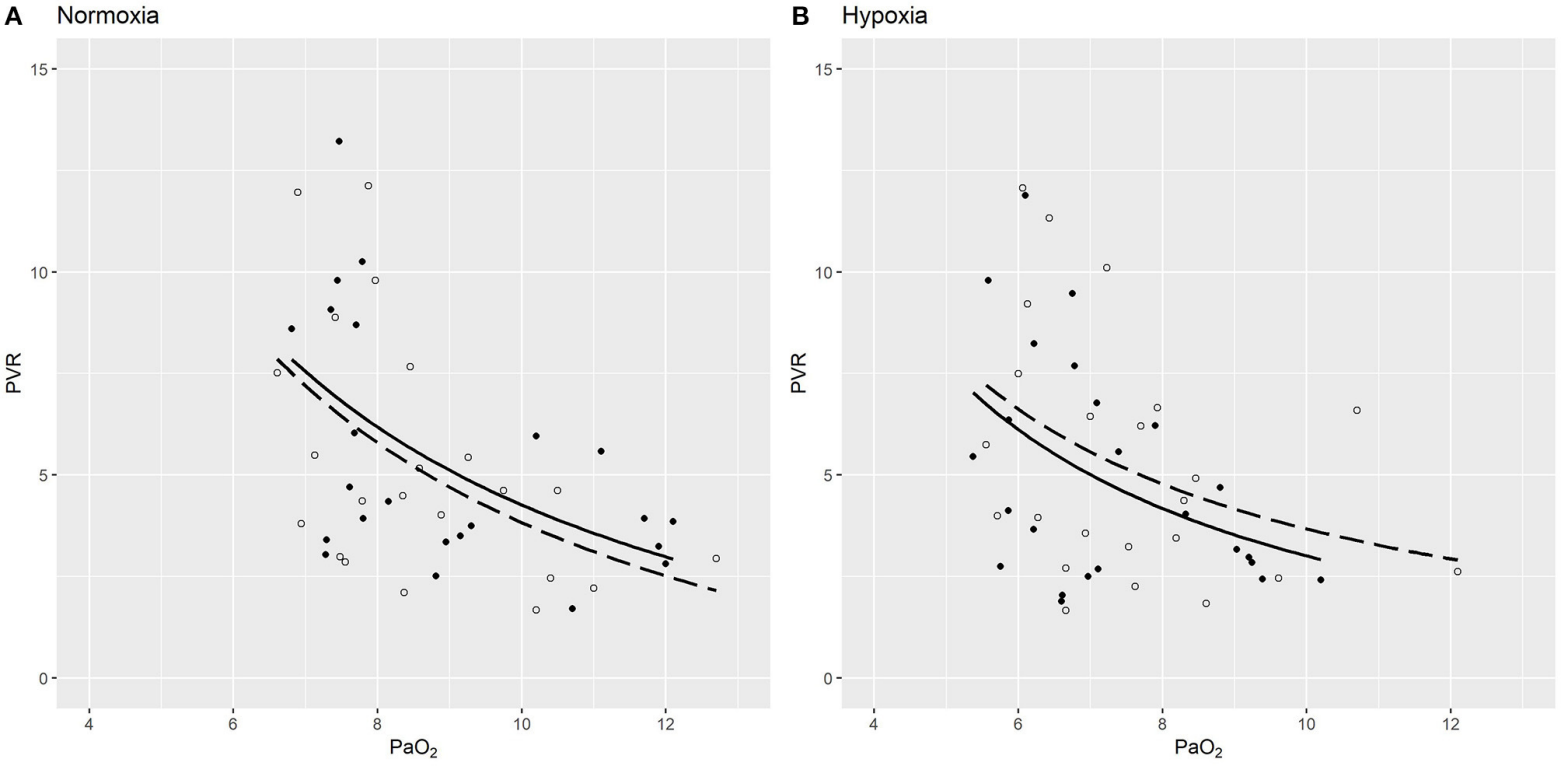
PVR (WU) plotted against PaO_2_ (kPa) dependent on treatment (acetazolamide vs. placebo) under normoxia **(A)** and hypoxia **(B)**. The light circles depict values under placebo saline treatment, while the filled circles show data points under acetazolamide treatment. Hyperbolic function curves (PVR ~ 1/PaO_2_) were fitted for placebo (dashed line) and acetazolamide (drawn out line) under each condition. In the hyperbolic mixed model, PVR inversely correlated with PaO2 (*p* = 0.006). Under normoxia **(A)** there was no significant difference in PVR induced by acetazolamide [0.21, 95-CI (−0.28 to 0.66), *p* = 0.354]. However, under hypoxia, a non-significant trend toward lower PVR is suggested in patients with acetazolamide treatment [mean change −0.41 WU, 95-CI (−0.93 to 0.11), *p* = 0.124), visualized by the downwards curve shift **(B)**.

The PVR measured after acetazolamide vs. placebo inversely correlated with PaO_2_ under both normoxic and hypoxic gas breathing (Figure 3). The hyperbolic response curve showed a downward displacement of the acetazolamide treatment curve compared to the placebo treatment curve under hypoxia, pointing toward an independent effect of acetazolamide treatment to decrease PVR under hypoxic conditions and is in line with the results from the mixed regression models. Exploratory analysis investigating the effect of the diagnostic group PAH or CTEPH on PVR or mPAP in the mixed multivariable regression model (including interventions, placebo or acetazolamide, normoxia or hypoxia, and the order of administration) did not reveal any significant difference on PVR or mPAP between CTEPH and PAH under both normoxia and hypoxia.

### Symptoms or Side Effects

No patient reported any carbonic anhydrase inhibitor-typical side effects such as paraesthesia, gastrointestinal distress, nausea, headache, or light headedness. Importantly, none described chest pain or dyspnea.

## Discussion

The present first randomized, double blind, placebo-controlled trial shows that 500 mg intravenous acetazolamide did not alter PVR measured after 1 hour by RHC and besides a slight increase in heart rate, other hemodynamic parameters were unaffected. Acetazolamide induced a slight metabolic acidosis, but neither PaCO_2_ nor PaO_2_ were significantly altered, so the known effects of renal carbonic anhydrase inhibition on ventilation and blood gases were not yet observed. The meaning of the decrease in PVR whilst breathing normobaric hypoxic gas (F_I_O_2_ 0.15) after acetazolamide but not placebo in regard of the unchanged PAP and CI remains to be clarified, it may represent some vasodilation or blunted hypoxic pulmonary vasoconstriction induced by acetazolamide under hypoxic conditions. Acute administration of acetazolamide did not induce adverse effects.

PH is a severe disorder with a dismal prognosis if left untreated, characterized by progressively elevated PVR leading to right ventricular failure. The pathobiology of PH is still incompletely understood but endothelial dysfunction, inflammation and vasoconstriction are all involved and lead to a severe pulmonary vasculopathy which might be shared over several distinct diagnostic groups. (29, 30) Although recent therapeutic advances including medical and interventional therapies have improved prognosis, PH-associated morbidity and mortality is still considerable and novel therapeutic strategies are highly warranted (31).

The carbonic anhydrase inhibitor acetazolamide stimulates ventilation and thus improves blood oxygenation and also possesses immune-modulatory effects with a potential to treat PH upon longer-term exposure (32–34). However, acetazolamide also prevents hypoxic pulmonary vasoconstriction independent of carbonic anhydrase inhibition, as shown in dogs, rodent models and isolated perfused lungs. (17–19, 21). In a prospective, double-blind cross-over trial in nine healthy, 3 days of preventive treatment with acetazolamide significantly reduced the rise in PAP during a hypoxia exposure, and a slightly decreased PVR was even observed in ambient air. (20, 21). This blunted response to acute hypoxia in healthy subjects was recently confirmed in 11 healthy young men treated for 2 days with oral acetazolamide, who had a smaller increase in systolic PAP compared to placebo whilst breathing normobaric hypoxic gas along with a decreased pulmonary vascular sensitivity to hypoxia. (35). In patients with COPD traveling to 3,100 m, the hypoxia-induced rise in PAP was significantly blunted by acetazolamide prophylaxis (36). In PH-patients tested during RHC, high-dose oxygen (F_I_O_2_ 1.0) decreased PAP, whereas PAP was not significantly altered with hypoxia (F_I_O_2_ 0.15) 0.3. (37). This may indicate that the already constricted pulmonary arteries of PH-patients demonstrate a blunted response to hypoxia, but still dilate with oxygen. The potential of acetazolamide to decrease pulmonary vasoconstriction in pulmonary vascular disease and the potential mode and duration of action are still unclear. In the present study, the PVR inversely correlated with the PaO_2_ as expected after both treatments. The hyperbolic response curve fitted for PVR vs. PaO_2_ showed a downward displacement of the acetazolamide treatment curve compared to the placebo treatment curve under hypoxia (Figure 3). This may indicate that PVR decreased independently of PaO_2_ under acetazolamide treatment under hypoxic conditions and is in line with the results from the mixed regression models. Results from rat models with hypoxia-induced PH revealed amelioration of PH after 10 and 17 days of acetazolamide treatment. (33, 38). In the present first study exploring the acute hemodynamic response 60 min after 500 mg intravenous acetazolamide in PH-patients, we could not demonstrate an acute pulmonary vasodilation after acetazolamide compared to placebo. Along with an unchanged PAP and CI, this study does not support the hypothesis of an acute vasodilator effect of acetazolamide after an hour under ambient air condition. Of interest, a significant reduction of the PVR along with a non-significant increase in CI and an unchanged PAP was found after the short exposure to normobaric hypoxia following acetazolamide, but not placebo-injection. Whether this might be interpreted as an early sign of vasodilation or blunted hypoxic pulmonary vasoconstriction by acetazolamide remains to be clarified by future research. HR was significantly increased after acetazolamide compared to placebo. The reason for that is not clear. Whereas carbonic anhydrase inhibition does not seem to affect HR, blood pressure and CO upon longer-term exposure, acute effects of acetazolamide on HR are insufficiently explored. In regard to a potential peripheral acute vasodilator effect as seen in the brain (39, 40), a small rise in HR seems conceivable. No further effects on other hemodynamic parameters were found. However, this study investigated the acute hemodynamic effect of acetazolamide after 60–90 min in patients with PH and results are thus not comparable to other studies which investigated healthy subjects after 2–3 days of oral acetazolamide intake (21, 35) and hemodynamic or even clinical responses to acetazolamide upon longer-term intake in PH-patients warrant further investigation.

In the present study, acetazolamide induced a slight metabolic acidosis reflected by a slightly lower arterial pH and bicarbonate after an hour of exposure in line with arterial blood gas results in a study investigating the cerebral blood flow after 30 and 60 min of 1 g intravenous acetazolamide which showed a comparable pH reduction after acetazolamide but no change in PaCO_2_. (39). However, in contrast to findings in the monocrotaline-hypoxia PH-rat models, where normocapnic acidosis induced for 5 days lowered right ventricular systolic pressure and reduced pulmonary arterial remodeling, the comparably small and acute reduction in pH in our PH-patients did not influence pulmonary hemodynamics. (32) Interestingly, PaCO_2_ was significantly lower in the placebo group during hypoxic gas breathing. One could argue, that we observed an immediate ventilatory response to the hypoxic conditions in the placebo group, whilst this response was blunted in the acetazolamide group possibly due to the slight improvement of hemodynamics. Thus, although we did not observe the typical respiratory stimulation that develops after longer-term acetazolamide intake that helps to prevent acute mountain sickness, our results confirm that the metabolic response with renal bicarbonate loss starts shortly after administration of intravenous acetazolamide, although the full response of a 4–6 mmol fall in plasma bicarbonate and its respiratory stimulation takes many hours to reach full effect. (39, 41) Accordingly, also in the current cohort, acetazolamide was associated with a very slight albeit significantly improved cerebral tissue oxygenation, most probably related to the previously described cerebral vasodilator effect of acetazolamide observed with 1,000 mg given i.v. (39), carbonic anhydrase is located in erythrocytes and the vascular endothelium and thus being ubiquitous, its inhibition is expected to increase CO_2_ concentrations in many tissues, including the brain, but potentially as well the lung. (42) In the brain, this effect is used diagnostically and therapeutically for vasodilation and treatment of epilepsy. (41) An increased cerebral perfusion and tissue oxygenation is of importance in many illnesses including sepsis or other critically ill states. (41) It has been shown that cerebral tissue oxygenation is a strong predictor of cognitive function and exercise performance in PH (43, 44). Whether carbonic anhydrase inhibition would affect lung vessels in analogy to brain vessels is not clear, preliminary studies, however, reveal that the blunted hypoxic pulmonary vasoconstriction by acetazolamide works through carbonic anhydrase independent mechanisms (45).

## Limitations

The crossover design allowed a reduction in the sample size in a rare disease such as PH, but has intrinsic limitations. We found no carry-over effect by including the treatment order in our regression models. A time lag of 60 min after acetazolamide vs. placebo injection was chosen to have a sufficiently long enough period to see an effect but still allow feasibility during RHC. Measures at the end of breathing normobaric hypoxic gas for 10–15 min were taken 80–90 min after acetazolamide vs. placebo, where the time lag might have influenced results.

In conclusion, this first randomized, placebo-controlled cross-over study in patients with PH shows for the first time that the PVR and other relevant hemodynamic parameters are not altered 1 h after 500 mg intravenous acetazolamide vs. placebo. The clinical meaning of a slightly but significantly decreased PVR during normobaric hypoxia breathing after acetazolamide but not placebo, resulting as combined effect of non-significantly elevated CI and unchanged mPAP, remains to be clarified. We found that acetazolamide slightly but significantly improved cerebral tissue oxygenation compared to placebo, most probably due to the known direct vasodilator effect induced by the lowered arterial pH and a direct carbonic anhydrase inhibition in brain vessels. Very importantly, patients tolerated the medication well with no reported side-effects or deleterious hemodynamic effects. These favorable safety results pave the way to study whether longer-term administration of acetazolamide will improve blood oxygenation and blunt pulmonary vasoconstriction through stimulated ventilation or other anti-inflammatory and anti-remodeling mechanisms, especially at night during sleep or exposure to hypoxia at altitude, and whether this will improve pulmonary hemodynamics and daytime performance.

## Data Availability Statement

The raw data supporting the conclusions of this article will be made available by the authors, without undue reservation.

## Ethics Statement

The studies involving human participants were reviewed and approved by ethics committee Zurich (BASEC 2016-00089). The patients/participants provided their written informed consent to participate in this study.

## Author Contributions

SU is the guarantor of the paper. All authors meet criteria for authorship as recommended by the International Committee of Medical Journal Editors. All authors contributed to the production of the final manuscript with revision for important intellectual content.

## Conflict of Interest

SU reports grants from Johnson and Johnson SA, Switzerland, during the conduct of the study; grants from Zurich Lung, grants from Orpha Swiss, personal fees from Actelion SA, Switzerland, personal fees from MSD Switzerland, outside the submitted work. The remaining authors declare that the research was conducted in the absence of any commercial or financial relationships that could be construed as a potential conflict of interest.
