# Supplementary material for: Acute Hemodynamic Effect of Acetazolamide in Patients With Pulmonary Hypertension Whilst Breathing Normoxic and Hypoxic Gas: A Randomized Cross-Over Trial
Source: Front Med (Lausanne). 2021 Jul 22;8:681473. doi: 10.3389/fmed.2021.681473 (PMC8341560; doi:10.3389/fmed.2021.681473)
Supplement: Supplementary file 1 [file Table_1.docx]

**Supplemental table 1: The effect of acetazolamide vs. placebo on invasive pulmonary hemodynamics assessed after 60 minutes under ambient air breathing at rest, adjusted for age, sex and respective baseline values**

| **Variable** | **Factor** | **Coefficients** | **95% CI** | | **p-value** |
| --- | --- | --- | --- | --- | --- |
| Pulmonary vascular resistance, WU | Acetazolamide | 0.19 | -0.20 | 0.57 | 0.341 |
|  | Age, y | 0.02 | -0.03 | 0.08 | 0.384 |
|  | Baseline values, WU | 1.19 | 0.87 | 1.51 | <0.001* |
|  | Female | 0.18 | -1.51 | 1.87 | 0.837 |
|  | Order (Acetazolamide first)  CTEPH vs PAH | -0.07  1.00 | -1.35  -0.76 | 1.22  2.76 | 0.920  0.265 |
| Mean pulmonary artery pressure, mmHg | Acetazolamide | 0.52 | -1.05 | 2.09 | 0.517 |
|  | Age, y | 0.02 | -0.16 | 0.20 | 0.848 |
|  | Baseline values, mmHg | 1.02 | 0.79 | 1.25 | <0.001* |
|  | Female | 1.17 | -4.28 | 6.62 | 0.673 |
|  | Order (Acetazolamide first)  CTEPH vs PAH | -0.51  3.17 | -4.60  -2.22 | 3.59  8.56 | 0.809  0.248 |
| Cardiac output, l//min | Acetazolamide | -0.16 | -0.56 | 0.23 | 0.423 |
|  | Age, y | -0.02 | -0.06 | 0.02 | 0.266 |
|  | Baseline values, l/min | 0.62 | 0.24 | 1.01 | 0.002* |
|  | Female | -0.40 | -1.48 | 0.69 | 0.475 |
|  | Order (Acetazolamide first)  CTEPH vs PAH | -0.20  -1.19 | -1.05  -2.16 | 0.65  -0.21 | 0.642  0.017* |
| Pulmonary artery wedge pressure, mmHg | Acetazolamide | -0.08 | -0.95 | 0.79 | 0.851 |
|  | Age, y | -0.05 | -0.13 | 0.04 | 0.264 |
|  | Baseline values, mmHg | 0.44 | 0.02 | 0.86 | 0.039* |
|  | Female | -0.77 | -3.15 | 1.61 | 0.525 |
|  | Order (Acetazolamide first)  CTEPH vs PAH | 1.53  -0.55 | -0.31  -3.09 | 3.37  2.00 | 0.104  0.675 |
| Heart rate, min^-1^ | Acetazolamide | 1.87 | 0.22 | 3.52 | 0.026* |
|  | Age, y | 0.20 | -0.05 | 0.45 | 0.113 |
|  | Baseline values, min^-1^ | 0.71 | 0.43 | 0.98 | <0.001* |
|  | Female | 5.52 | -2.26 | 13.30 | 0.165 |
|  | Order (Acetazolamide first)  CTEPH vs PAH | -2.86  5.62 | -9.02  -2.44 | 3.31  13.68 | 0.364  0.172 |
| pH | Acetazolamide | -0.01 | -0.02 | -0.01 | <0.001* |
|  | Age, y | 0.00 | -0.00 | 0.00 | 0.567 |
|  | Baseline values | 0.41 | 0.13 | 0.70 | 0.005* |
|  | Female | -0.01 | -0.03 | 0.01 | 0.441 |
|  | Order (Acetazolamide first)  CTEPH vs PAH | -0.01  0.01 | -0.03  -0.02 | 0.01  0.03 | 0.390  0.609 |
| Cerebral tissue oxygenation, % | Acetazolamide | 2.39 | 0.42 | 4.35 | 0.017* |
|  | Age, y | 0.00 | -0.11 | 0.11 | 0.964 |
|  | Baseline values, % | 0.72 | 0.51 | 0.94 | <0.001* |
|  | Female | 0.93 | -2.33 | 4.19 | 0.578 |
|  | Order (Acetazolamide first)  CTEPH vs PAH | -0.05  1.58 | -2.58  -1.59 | 2.47  4.75 | 0.967  0.329 |

Data calculated from mixed linear regression models are adjusted for age, respective baseline values, sex, treatment order and the study drug (acetazolamide vs. placebo). Values of the respective variable and displayed as mean ± SD. * indicates statistical significance between placebo-saline and acetazolamide in the randomized phase.
