# Supplementary material for: Acute Hemodynamic Effect of Acetazolamide in Patients With Pulmonary Hypertension Whilst Breathing Normoxic and Hypoxic Gas: A Randomized Cross-Over Trial
Source: Front Med (Lausanne). 2021 Jul 22;8:681473. doi: 10.3389/fmed.2021.681473 (PMC8341560; doi:10.3389/fmed.2021.681473)
Supplement: Supplementary file 2 [file Table_2.docx]

**Supplemental table 2: The effect of acetazolamide vs. placebo on hypoxia-induced change in invasive pulmonary hemodynamics, adjusted for age, sex and baseline values**

| Variable | Factor | Coefficients | 95% Confidence Interval | | P value |
| --- | --- | --- | --- | --- | --- |
| Pulmonary vascular resistance, WU | Acetazolamide | -0.45 | -0.88 | -0.01 | 0.046* |
|  | Age, y | 0.00 | -0.02 | 0.03 | 0.927 |
|  | Baseline values, WU | 0.01 | -0.11 | 0.13 | 0.877 |
|  | Female | 0.58 | -0.18 | 1.35 | 0.135 |
|  | Order (Acetazolamide first)  CTEPH vs PAH | 0.09  -0.45 | -0.49  -1.26 | 0.68  0.36 | 0.752  0.275 |
| Mean PAP, mmHg | Acetazolamide | -0.42 | -3.25 | 2.41 | 0.769 |
|  | Age, y | -0.03 | -0.19 | 0.13 | 0.713 |
|  | Baseline values, mmHg | 0.14 | -0.04 | 0.32 | 0.116 |
|  | Female | 3.12 | -1.74 | 7.97 | 0.208 |
|  | Order (Acetazolamide first)  CTEPH vs PAH | 0.33  -1.56 | -3.39  -6.70 | 4.05  3.58 | 0.861  0.552 |
| Cardiac output, l/min | Acetazolamide | 0.35 | -0.09 | 0.80 | 0.118 |
|  | Age, y | 0.00 | -0.02 | 0.02 | 0.924 |
|  | Baseline values, l/min | -0.10 | -0.29 | 0.09 | 0.297 |
|  | Female | 0.20 | -0.38 | 0.78 | 0.506 |
|  | Order (Acetazolamide first)  CTEPH vs PAH | 0.06  0.04 | -0.39  -0.54 | 0.51  0.62 | 0.789  0.889 |
| Pulmonary artery wedge pressure, mmHg | Acetazolamide | 0.13 | -0.85 | 1.10 | 0.802 |
|  | Age, y | -0.03 | -0.10 | 0.04 | 0.359 |
|  | Baseline values, mmHg | 0.45 | 0.16 | 0.74 | 0.002* |
|  | Female | -0.52 | -2.58 | 1.54 | 0.621 |
|  | Order (Acetazolamide first)  CTEPH vs PAH | 0.23  -0.10 | -1.43  -1.57 | 1.89  1.38 | 0.785  0.897 |
| Heart rate, min^-1^ | Acetazolamide | -1.05 | -2.98 | 0.87 | 0.283 |
|  | Age, y | -0.23 | -0.36 | -0.94 | 0.001* |
|  | Baseline values, min^-1^ | -0.01 | -0.15 | 0.12 | 0.838 |
|  | Female | -1.33 | -5.65 | 3.00 | 0.548 |
|  | Order (Acetazolamide first)  CTEPH vs PAH | 1.02  -3.43 | -2.28  -7.98 | 4.33  1.13 | 0.544  0.140 |
| pH | Acetazolamide | -0.01 | -0.02 | 0.00 | 0.102 |
|  | Age, y | -0.00 | -0.00 | 0.00 | 0.206 |
|  | Baseline values | 0.54 | 0.15 | 0.94 | 0.007* |
|  | Female | -0.01 | -0.03 | 0.02 | 0.680 |
|  | Order (Acetazolamide first)  CTEPH vs PAH | 0.01  0.02 | -0.02  -0.01 | 0.03  0.05 | 0.657  0.155 |
| CTO, % | Acetazolamide | -0.98 | -2.71 | 0.75 | 0.268 |
|  | Age, y | 0.00 | -0.08 | 0.09 | 0.940 |
|  | Baseline values, % | 0.22 | 0.03 | 0.40 | 0.023* |
|  | Female | 1.18 | -1.48 | 3.83 | 0.385 |
|  | Order (Acetazolamide first)  CTEPH vs PAH | -2.61  1.29 | -4.66  -1.28 | -0.56  3.86 | 0.013*  0.326 |

Data calculated from mixed linear regression models are adjusted for treatment order, age, sex and baseline values of the respective variable and displayed as mean ± SD. * indicates statistical significance between placebo-saline and acetazolamide in the randomized phase.
